# Immune regulation: a new strategy for traditional Chinese medicine-based treatment of granulomatous lobular mastitis

**DOI:** 10.3389/fimmu.2024.1494155

**Published:** 2024-10-31

**Authors:** Yuan Lou, Han Xu, Zixuan Lu, Bin Wang, Xiaofei Liu

**Affiliations:** ^1^ First Clinical Medical College, Shandong University of Traditional Chinese Medicine, Jinan, Shangdong, China; ^2^ Department of Immunology, Binzhou Medical University, Yantai, Shandong, China; ^3^ Breast and Thyroid Surgery, Affiliated Hospital of Shandong University of Traditional Chinese Medicine, Jinan, Shangdong, China

**Keywords:** granulomatous lobular mastitis, autoimmunity, pathogenesis, traditional Chinese medicine, pyroptosis, crosstalk

## Abstract

Granulomatous lobular mastitis (GLM) presents significant challenges, including high rates of morbidity, recurrence, and disability, ultimately impacting women’s health and quality of life. Local autoimmune imbalance involving dysregulated cytokines and immune cells has been recognized to play a key role in the pathology of GLM. Traditional Chinese medicine (TCM), with its multi-component, multi-pathway and multi-target characteristics, offers unique advantages and broad prospects in the treatment of GLM. Here, we review the relationship between immune dysregulation and GLM, as well as the regulatory mechanisms of TCM-based interventions, with the aim of providing new insights and foundational knowledge for the clinical treatment of GLM, while promoting the further application and development of TCM-based strategies for the treatment of GLM.

## 1 Introduction

Granulomatous lobular mastitis (GLM) is a distinct form of inflammatory breast disease, characterized by non-caseating necrosis of breast lobules and granuloma formation, accounting for 24% of all cases of inflammatory breast disease (1). GLM typically presents with a wide range of lesions, a prolonged course, and a high recurrence rate, severely impacting the physical and mental health, as well as the quality of life of patients (2). Current treatment options include hormone therapy, immunosuppressants, and localized surgical interventions. However, these approaches are often associated with extended treatment durations of 3 to 12 months, extensive surgeries required to achieve complete lesion removal, and notably, recurrence rates ranging from 10% to 50% (3). Therefore, finding effective therapies and drugs for treating GLM is an urgent clinical issue to be addressed.

Immune imbalance plays pivotal roles in the onset and progression of autoimmune diseases. Aberrantly activated immune cells release large amounts of cytokines, triggering autoimmune attacks on host tissues. This overexpression of cytokines further exacerbates immune system hyperactivity, creating a cycle that worsens disease severity. Studies have demonstrated that in GLM, dysregulation of T cells, B cells, and Natural killer (NK) cells, along with elevated release of inflammatory cytokines, are central to the disease process. These immune dysregulations can be alleviated by immunosuppressive treatments, suggesting that immune imbalance is a key mechanism underlying the pathological damage in GLM (2, 4, 5).

Traditional Chinese Medicine (TCM), known for its multi-component, multi-pathway, and multi-target approach, has demonstrated remarkable clinical efficacy in the treatment of GLM (6–8). Although the exact mechanisms by which TCM treats GLM remain unclear, its ability to restore the local immune microenvironment at the lesion site plays a critical role (9–11). In this review, we summarize the immune-mediated pathological mechanisms of GLM and the role of TCM in recovering local immune microenvironment, with the aim of advancing the application and research of TCM in the prevention and treatment of GLM.

## 2 Immune dysregulation and GLM

### 2.1 Macrophages

**Figure 1:**
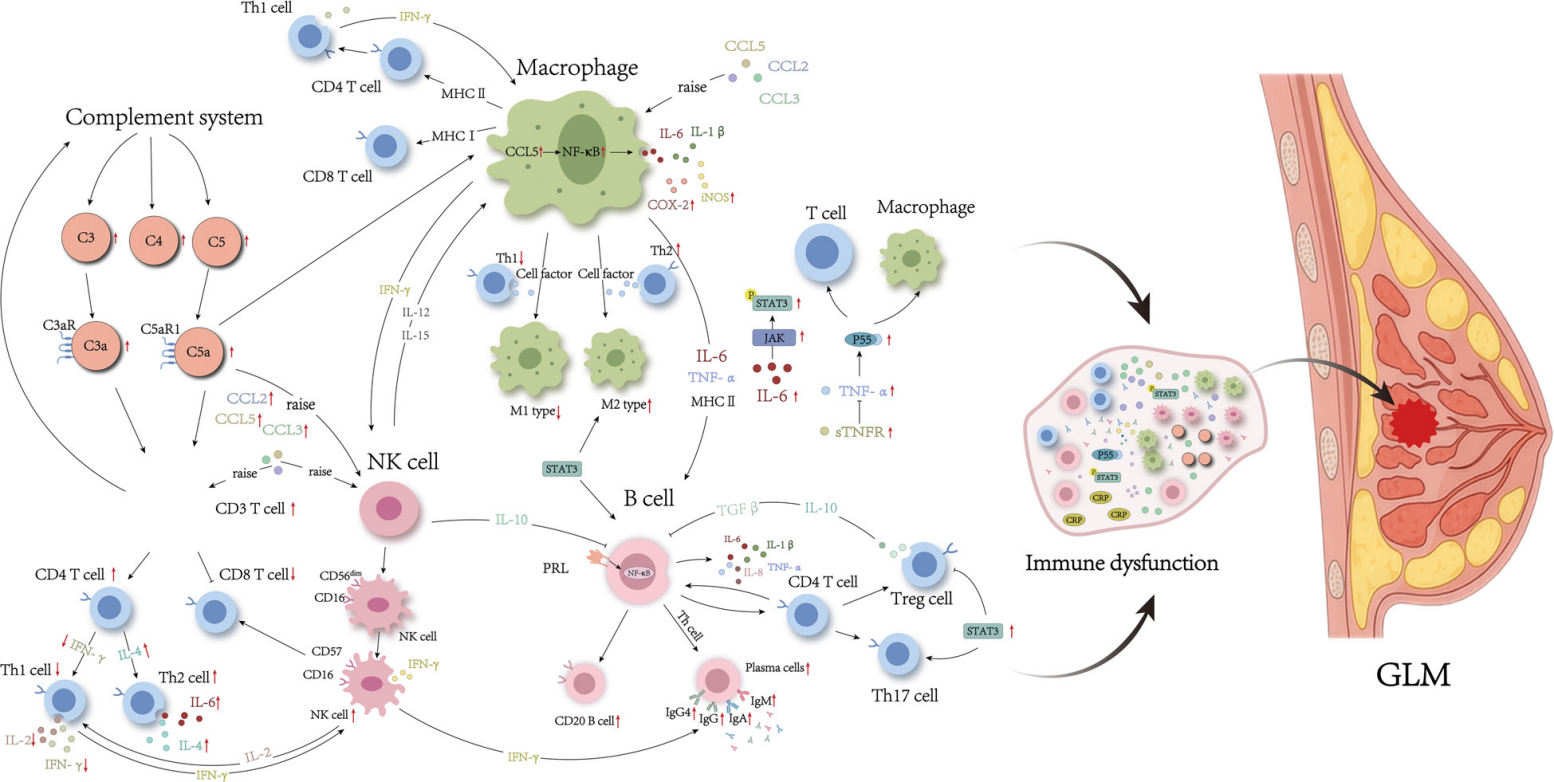
Immune disorders in GLM lesions. The red arrow indicates that experimental results have been validated.

Granuloma formation is the hallmark pathological feature of GLM, with macrophage infiltration and proliferation being directly responsible for inducing granuloma development (12). Macrophages, the most widely distributed immune cells in human tissues and the first line of defense against invaders, play a crucial role in GLM by releasing inflammatory factors and modulating the M1/M2 phenotype (13) (**Figure 1**). During the progression of GLM, the persistent secretion and activation of pro-inflammatory cytokines, including interleukin (IL)-1β and IL-6, promote macrophage chemotaxis and ongoing tissue destruction (11, 14, 15). Within macrophages, the nuclear factor-κB (NF-κB) pathway regulates the release of various inflammatory factors, including IL-6, IL-1β, cyclooxygenase-2 (COX-2), and inducible nitric oxide synthase (iNOS) (16, 17). Wang et al. (14) found significantly increased expression levels of phosphorylated p65, iNOS, and COX-2 in lesions of patients with GLM, closely linked to NF-κB pathway activation induced by C-C motif chemokine ligand (CCL)5 in macrophages.

Macrophage polarization refers to the different activated states of macrophages under specific conditions, categorized into pro-inflammatory M1 and anti-inflammatory M2 types based on interactions with T helper (Th) cells (18). M1 macrophages are activated by Th1 cells to participate in Th1-type immune responses, releasing pro-inflammatory factors that contribute to early-stage tissue damage and inflammation (19). In contrast, M2 macrophages, activated by cytokines secreted by Th2 cells, primarily exert anti-inflammatory effects by releasing anti-inflammatory cytokines, growth factors, and repair factors, promoting immune tolerance, tissue repair, and in some cases, tumorigenic activities (20). Studies have identified M2 macrophage infiltration in patients with GLM, suggesting that during the chronic phase, preventing the transition from M1 to M2 macrophages or inhibiting M2 macrophage activity could suppress tumorigenic effects, reduce the extent of surgery needed, and minimize post-GLM tissue damage (20).

### 2.2 T cells

**Figure 2:**
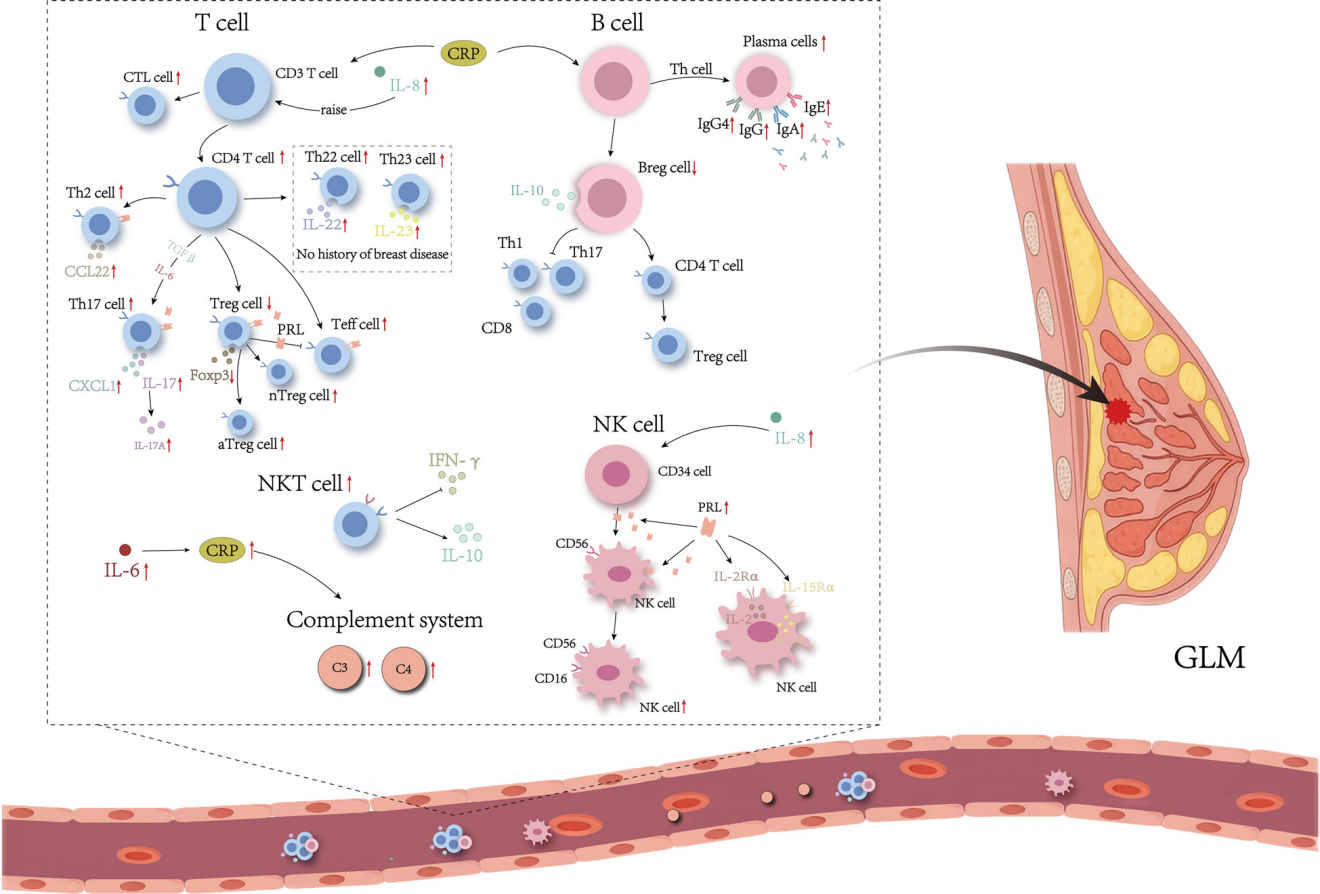
Immune disorders in serum from patients with GLM. The red arrow indicates that experimental results have been validated.

Numerous studies have recognized the remarkably disordered T cell subsets in GLM lesion (**Figures 1**, **2**). For example, immunohistochemical staining has consistently identified an inflammatory microenvironment predominantly composed of T cells in individuals with GLM (21, 22). Moreover, multiple subtypes of Th cells are abundantly expressed in the serum and lesion sites of patients with GLM (11, 23–25). Zheng et al. (21) provided a comprehensive review of the role of CD4^+^T cell subsets in GLM pathogenesis. Among these subpopulations, the Th1/Th2 imbalance has been strongly implicated in GLM development (21). In general, Th1 and Th2 maintain a dynamic equilibrium, but pathological conditions can cause a shift, with one subgroup dominating. In GLM, studies have shown an increase in IL-4 and IL-6 mediated by Th2 cells, alongside a decrease in IL-2 and interferon-γ (IFN-γ) mediated by Th1 cells, indicating a Th2-dominant inflammatory response (11, 21, 25). Furthermore, the recently identified pathogenic Th2 (Tpath2) cells have been shown to express high levels of ST2, a component of the IL-33 receptor, and IL-5 when activated by IL-33 (26, 27). The elevated IL-5 level can exacerbate the eosinophilic inflammation observed in GLM (28). However, the role of Tpath2 cells in GLM remains largely unexplored and warrants further investigation.

Regulatory T (Treg) cells are a CD4^+^ T cell subset with immunosuppressive functions, playing crucial roles in maintaining immune tolerance (29). The transcription factor forkhead box protein P3 (Foxp3) is specifically expressed in Treg cells. In contrast, effector T (Teff) cells, which proliferate and differentiate from naive CD4^+^T cells, have effects opposite to those of Treg cells, and maintaining a balance between Treg and Teff cells is essential for preventing autoimmune diseases (29). Legorreta-Haquet et al. (30) found that both Treg and Teff cells express high levels of prolactin (PRL) in patients with autoimmune disease. PRL binds to its receptor PRLR, reducing the inhibitory effect of Treg cells on Teff cells, consequently promoting Teff cell proliferation, increasing IFN-γ secretion, and exacerbating autoimmunity. Ucaryilmaz et al. (31) demonstrated that Teff cells are significantly elevated in the peripheral blood of patients with GLM compared to healthy controls, whereas Foxp3 expression in Treg cells and nTreg (CD3^+^CD4^+^CD45RA^−^Foxp3^low^) expression are lower in patients with GLM compared to healthy individuals. Furthermore, the proportion of aTreg (CD3^+^CD4^+^CD45RA^−^Foxp3^high^) and nTreg cells is higher during the active phase compared to the remission phase, suggesting that Teff/Treg immune imbalance and Foxp3 involvement contribute to the progression of GLM, with Treg cell subset differentiation varying across different pathological stages. On the other hand, under the influence of transforming growth factor β (TGF-β) and IL-6, Th0 cells differentiate into Th17 cells, which are involved in inflammatory responses and autoimmune diseases. Studies have indicated that the Th17/Treg balance is also crucial in maintaining immune homeostasis, as these cells are usually in dynamic equilibrium, mutually inhibiting each other (21). Koksal et al. (23) found significantly elevated levels of the Th17 characteristic cytokine IL-17 in the peripheral blood of patients with GLM, indicating Th17 involvement in GLM disease progression. Th17/Treg immune imbalance may be another important mechanism underlying GLM pathogenesis.

However, Saydam et al. (24) found that IL-17 levels in peripheral blood of premenopausal GLM patients without a history of breast disease did not differ significantly from those in healthy controls, whereas the levels of Th22 and Th23 characteristic cytokines IL-22 and IL-23 were significantly higher than in healthy individuals. Notably, evidence suggests that IL-23 is crucial for Th17 cell growth and can directly stimulate Th17 cells to produce IL-17 (32). Additionally, hyperprolactinemia has been shown to mediate changes in immune cells such as Th1, Th2, and Th17 (33). Ewerman et al. (34) found that the levels of Th2 and Th17-related factors CCL22 and CXC motif chemokine ligand (CXCL) 1 were significantly elevated in patients with mild to moderate hyperprolactinemia (465-3600 mU/I) compared to healthy controls.

In addition, cytotoxic T lymphocytes (CTLs) and natural killer T cells (NKTs) have been deeply implicated in granuloma formation. NKT cells inhibit yeast glycan A-induced granuloma formation by inhibiting IFN-γ production and promoting IL-10 production, which in turn inhibits yeast glycan A-induced granuloma formation, whereas CTL cells accumulate during the effector phase of granuloma formation (35). Emsen et al. (36) showed that the absolute CTL cell counts, absolute NKT cell counts and percentages in the peripheral blood of patients with GLM were significantly higher than those of patients in remission. In brief, the distribution of T cell subtypes varies in serum and focal tissues of patients with GLM and in different histories and pathological stages. However, the precise mechanisms by which T cell subtypes influence GLM progression remain unclear and require further investigation.

### 2.3 B cells

B cells play critical roles in the initiation, progression, and persistence of autoimmune diseases (**Figures 1**, **2**). Studies have demonstrated that B cells contribute to the development and advancement of GLM through the regulation of cytokine and antibody release, as well as the differentiation of regulatory B cells (Bregs). Li et al. (22) found that peripheral CD20^+^B cells in GLM lesions significantly outnumber CD3^+^T cells. The PRL-induced B-cell NF-κB signaling pathway in mammary epithelial cells can upregulate the pro-inflammatory cytokines in mammary epithelial cells. Research indicates elevated levels of IL-6 and tumor necrosis factor-α (TNF-α) in lesion tissues of patients with GLM (11). Activated B cells can regulate the function and migration of dendritic cells, macrophages, and T cells by secreting pro-inflammatory cytokines such as IL-6 and TNF-α, thereby providing feedback stimulatory signals that further activate B cells (37, 38).

Bregs play vital roles in maintaining immune tolerance, restoring immune homeostasis, and suppressing autoimmune-mediated inflammatory responses (39). IL-10^+^Breg cell can suppress pro-inflammatory responses from Th1, Th17, CD8^+^ T cells, NK cells, and dendritic cells, while also reducing the secretion of inflammatory factors. Additionally, they can convert naïve CD4^+^T cells into Treg cells (40). Tang et al. (41) demonstrated low expression of IL-10 in the serum of patients with GLM. Ucaryilmaz et al. (31) observed that patients with active GLM had a lower proportion of Breg cells in their peripheral blood compared to both patients in remission-phase GLM and healthy individuals. Therefore, increasing Breg cell expression and IL-10 release may be effective strategies for treating GLM and alleviating ongoing immune-mediated inflammatory damage.

After receiving antigen stimulation, mature B cells differentiate into plasma cells with the assistance of antigen-presenting cells and Th cells, thereby participating in humoral immunity. Research has found elevated levels of immunoglobulin (Ig) A, IgG, and IgM in the pus tissue and serum of patients with GLM (41, 42). Moreover, various Th cell subtypes are widely expressed in the serum and lesions of patients with GLM (11, 23–25). IgG4, secreted by plasma cells, is a Th2 cell-dependent immunoglobulin. Ogura et al. (43) found that patients with GLM can be categorized into IgG4-related and non-IgG4-related types. Patients with IgG4-related GLM exhibit diffuse IgG4^+^ plasma cells and elevated serum IgG4 levels, independent of pregnancy or childbirth factors, while non-IgG4-related GLM may be associated with these factors. Kong et al. (20) reported that IgG4 expression in patients with GLM varies based on the presence of nipple retraction: patients with nipple retraction show significantly higher expression of IgG4 in breast tissue compared to those without nipple retraction, suggesting that the increase in IgG4 may be related to duct obstruction, damage to ductal endothelial cells, and nipple inversion.

### 2.4 NK cells

NK cells are vital components of innate immunity. They participate in immune regulation through cell cytotoxicity and cytokine secretion functions (44, 45). Previous studies have shown that NK cells are involved in the disease progression of GLM (**Figures 1**, **2**). Research has shown that the absolute count and proportion of CD16^+^CD56^+^NK cells in the peripheral blood of patients with active GLM are significantly increased (36). On one hand, CD57 is mainly expressed by the mature subset of NK cells. The peak infiltration of CD57^+^NK cells in the breast tissue of patients with GLM at the chronic phase (2 weeks to 3 months) may indicate that CD57^+^NK cell levels serve as an important clinical boundary for GLM (20). In addition, compared to CD57^-^NK cells, CD57^+^NK cells produce higher levels of IFN-γ that may further activate M1-type macrophages (44). On the other hand, NK cells express PRLR, which has been identified to mediate and promote the differentiation of CD56^+^ NK cells from the other CD34^+^ lineage (46, 47). Research has demonstrated that PRL induces the upregulation of IL-2/IL-2Rα and IL-15/IL-15Rα expression in an autocrine manner, activating the cytotoxicity of NK cells. Therefore, regulating the function of NK cells or the cytokines that stimulate and inhibit their secretion could be crucial for the clinical alleviation or cure of GLM.

### 2.5 Complement system

The complement (C) system is an important component of the innate immune system, playing a central role in the fight against pathogens and inflammation. Evidence suggests that the complement system is involved in the pathogenesis of GLM. Su et al. (48) found elevated levels of C3 and C4, intrinsic components of complement, in the serum and diseased tissues of patients with GLM, with their levels decreasing after treatment. C3 and C5 belong to the complement system and mediate a various autoimmune diseases (49, 50). During inflammatory stimulation, C3 and C5 are cleaved by proteolytic processes into the chemokines C3a and C5a, which then bind to their respective receptors, C3aR and C5aR1. This interaction promotes histamine release from mast cells and basophils, and stimulates the recruitment of polymorphonuclear leukocytes to the site of inflammation, contributing to the regulation of the inflammatory response (51). Li et al. (49) reported that C3a and C5a were highly expressed in exosomes from the tissues of GLM patients, along with elevated levels of C3/C3a-C3aR and C5/C5a-C5aR1. This suggests that the complement system is activated in GLM, and that inflammation-stimulated production of C3a and C5a is enriched in exosomes, participating in GLM pathogenesis by binding to their receptors and acting as paracrine factors (**Figures 1**, **2**).

### 2.6 Autoantibodies

The immune response involving autoantibodies targeting self-cells or components is a primary cause of pathological damage in autoimmune diseases and serves as a key indicator for disease diagnosis and prognosis. Ozel et al. (52) conducted autoantibody tests for rheumatoid factor (RF), anti-nuclear antibodies (ANA), and anti-double-stranded DNA (anti-dsDNA) antibodies on eight patients diagnosed with GLM who underwent surgical treatment. The results indicated that all autoantibodies were positive in 3 patients, RF was positive in 6 patients, and 2 patients were positive for both ANA and anti-dsDNA antibodies. Shojaeian et al. (53) tested 318 patients diagnosed with refractory or recurrent GLM, revealing that 11.6% of the entire patient cohort tested positive for ANA. Following treatment with methotrexate, a drug indicated for rheumatoid arthritis, combined with low-dose corticosteroids, 94.3% of patients achieved complete remission. However, Koksal (54) reported that the positivity rates of RF, ANA, anti-dsDNA antibodies, perinuclear anti-neutrophil cytoplasmic antibodies (pANCA), and anti-cyclic citrullinated peptide antibodies (anti-CCP) in patients with active GLM showed no statistically significant difference compared to healthy females. Therefore, further clinical data are needed for validation.

In summary, as an autoimmune disease, the pathogenesis of GLM is associated with T cells, B cells, macrophages, NK cells, the complement system, autoantibodies, and cytokines to varying degrees. These factors interact and collectively exacerbate GLM damage. On one hand, cellular immunity, represented by T cells, promotes changes in the immune microenvironment in GLM by recognizing, binding to, and presenting antigens, assisting humoral immunity, and releasing cytokines, thereby triggering an immune storm. On the other hand, humoral immunity, represented by B cells, releases inflammatory factors and antibodies in GLM and regulates immune cell activity, further promoting inflammation and damage. Notably, the state of immune dysregulation in patients with GLM varies across different pathological stages, disease histories, peripheral blood, and lesion tissues. Evidence suggests that the level of autoreactive antibodies increases following B cell senescence, contributing to the onset and progression of autoimmune diseases (55, 56). In multiple autoimmune diseases, the number of autoreactive and age-related B cells is significantly higher in patients and autoimmune-prone mice compared to healthy controls (57).

Additionally, cytokines such as IL-8, IL-17A, and CCL2 also contribute to the onset and progression of GLM. Specifically, TNF-α plays a critical role in recruiting immune cells involved in granuloma formation through its chemotactic effects (58). In the early stages of GLM, large amounts of TNF-α released bind to receptor P55, thereby promoting inflammation and immune cell recruitment (59). Wang et al. (14) used cytokine chip technology to analyze changes in inflammatory cytokines in patients with GLM and found elevated CCL2, CCL3, CCL5, and soluble TNF receptor (sTNFR) expression levels in lesion tissues. Moreover, evidence indicates that both serum IL-6 and C-reactive protein (CRP), along with phosphorylated STAT3 (pSTAT3) in lesion sites, are elevated in patients with GLM. This may result from IL-6 functioning as a CRP-inducing factor, promoting its synthesis and release which under immune stimulation, which leads to the overactivation of the Janus kinase/signal transducer and activator of transcription 3 (JAK/STAT3) pathway (15, 60). Additionally, PRL induces a dose-dependent amplification of mRNA expression for pro-inflammatory cytokines such as IL-1β, IL-6, IL-8, granulocyte-macrophage colony-stimulating factor (GM-CSF), and TNF-α in mammary epithelial cells, subsequently triggering inflammation and granuloma formation in breast tissues (61) (**Figures 1**, **2**). This suggests that cytokines synthesized and secreted by immune cells are extensively involved in the pathogenesis and progression of GLM. However, the role of these cytokines in mediating the development, activation, and targeted migration of immune cells in this process remains unclear. Therefore, further investigation into the pathogenic mechanisms of GLM is necessary to establish more accurate diagnostic modalities and more effective therapeutic strategies to address the challenges posed by varying pathological stages and individual differences.

## 3 Treatment of GLM with TCM through immune regulation

Based on clinical symptom manifestations, GLM is classified in TCM as belonging to the category of “mammary carbuncle”. Based on the etiology, pathogenesis, and clinical characteristics, GLM is classified in TCM into three different stages: the lump phase, abscess stage, and late stage of ulceration. The goals of treatment include soothing the liver, clearing heat, reducing swelling, relieving pain, detoxification, clearing heat, promoting suppuration drainage, resolving carbuncle, and supporting the body’s resistance while expelling toxins. The therapeutic mechanisms of TCM in treating GLM have been well reviewed by Lian et al (8, 62). Research has demonstrated that TCM treatments can significantly improve breast shape in patients with GLM, promote wound healing, shorten the disease course, alleviate pain, reduce recurrence rates, and enhance the overall quality of life without significant toxic side effects, indicating high safety and efficacy (8, 62).

Recently, research on the immunomodulatory mechanisms of TCM-based interventions has gained significant attention. Studies have shown that systemic treatment with Chinese herbal medicine modulates immune pathways in the treatment of GLM (**Table 1**). Chaihu Qinggan Decoction, derived from “surgical authentic” formulas, is recognized for its effects in soothing the liver, relieving depression, clearing heat, detoxifying, and reducing swelling. It is commonly applied in the clinical management of the acute progression phase of GLM (63). Combining Chaihu Qinggan Decoction with prednisone significantly increases the expression of CD3^+^, CD4^+^, and complement CD3 in peripheral blood while reducing CD8^+^, IgG, IgA, and IgM expression levels, thus improving therapeutic efficacy and regulating immune function (63). Xie et al. (63) explored the mechanisms by which Chaihu Qinggan Decoction treats GLM using network pharmacology. Their findings indicated that active ingredients such as quercetin, kaempferol, and baicalein may target IL-6, TNF, IL-1β, CXCL8, CCL2, and IL-4, and modulate IL-17, Toll-like receptor, and TNF-related pathways. These activities could impact M1/M2 macrophage polarization, thereby regulating downstream inflammatory factors and influencing GLM progression. Other TCM formulas such as Shugan Xiaoyong Decoction and Tuoli Tounong Decoction also mitigate autoimmune inflammatory damage through immunomodulation, providing therapeutic benefits for patients with GLM (64, 65).

**Table 1:** TCM treats GLM through immunoregulation.

Classification	Methods	Composition/Source	Immunomodulation Or Other regulation	Ref
**Internal treatment method**	Shugan Xiaoyong Decoction	Benincasae Semen, Taraxaci Herba, Poria, Coicis Semen, Rhodiolae Crenulatae Radix et Rhizoma, Forsythiae Fructus, Hordei Fructus Germinatus, Smilacis Chinae Rhizoma, Actinidia arguta (Sieb. & Zucc) Planch., Paeoniae Radix Rubra, Arnebiae Radix, Prunellae Spica, Bupleuri Radix (prepared with vinegar), Curcumae Radix, Fritillariae Thunbergii Bulbus, Angelicae Dahuricae Radix, Cimicifugae Rhizoma, Glycyrrhizae Radix et Rhizoma	Downregulation of WBC, CRP, IL-2 expression, upregulation of IL-4 expression.	(64)
	Soothing the liver and requlating Qi, removing blood stasis and dredging Decoction	Bupleuri Radix, Lonicerae Japonicae Flos, Forsythiae Fructus, Platycodonis Radix, Aurantii Fructus, Trichosanthis Radix, Bombyx Batryticatus, Trichosanthis Fructus, Fritillariae Thunbergii Bulbus, Polygoni Cuspidati Rhizoma et Radix, Angelicae Sinensis Radix, Chuanxiong Rhizoma, Paeoniae Radix Rubra, Paeoniae Radix Alba, Prunellae Spica, Glycyrrhizae Radix et Rhizoma	Downregulation of Ki-67 antigen expression.	(68)
	Yiqi Heying Decoction	Astragali Radix, Codonopsis Radix, Bupleuri Radix, Angelicae Sinensis Radix, Salviae Miltiorrhizae Radix et Rhizoma, Paeoniae Radix Rubra, Taraxaci Herba, Forsythiae Fructus, Scutellariae Radix, Polygoni Cuspidati Rhizoma et Radix, Gleditsiae Spina, Glycyrrhizae Radix et Rhizoma	Downregulation of IL-6 and IL-1β, C3, C4, CRP, IgA, IgG, IgM expression, upregulation of IFN-γ expression.	(11, 42, 48)
	Chaihu Qinggan decoction	Bupleuri Radix, Angelicae Sinensis Radix, Chuanxiong Rhizoma, Rehmanniae Radix, Paeoniae Radix Rubra, Scutellariae Radix, Trichosanthis Radix, Saposhnikoviae Radix, Arctii Fructus, Gardeniae Fructus, Forsythiae Fructus, Glycyrrhizae Radix et Rhizoma	Upregulation of CD3^+^ and CD4^+^, expression, downregulation of CD8^+^, IgG, IgA, IgM, IL-1, IL-6, TNF-α and PRL expression; Downregulation of NLRP3, Caspase-1, and IL-1β expression.	(9, 69, 70)
	Xihuang Capsule	Bovis Calculus, Olibanum, Myrrha, Notoginseng Radix et Rhizoma, Panacis Quinquefolii Radix, Cremastrae Pseudobulbus Pleiones Pseudobulbus, Cordyceps, Margaritifera Concha, Moschus	Downregulation of BAX, IL-6, JAK2, STAT3, Caspase-9 expression. Downregulation of TNF-α, BAX, IL-1β, NF-κB expression.	(71, 72)
	Tuoli Tounong Decoction	Ginseng Radix et Rhizoma, Atractylodis Macrocephalae Rhizoma, Vaccariae Semen, Angelicae Dahuricae Radix, Cimicifugae Rhizoma, Glycyrrhizae Radix et Rhizoma, Angelicae Sinensis Radix, Astragali Radix, Gleditsiae Spina, Citri Reticulatae Pericarpium Viride	Downregulation of IL-18 and IL-1β, Caspase-1 and GSDMD-N expression.	(65)
	Tounong Powder	Astragali Radix, Angelicae Sinensis Radix, Chuanxiong Rhizoma, Manis, Gleditsiae Spina	Downregulation of IL-6, TNF, IL-1β, IL-2, IL-4, and CRP expression.	(73, 74)
	Yanghe Decoction	Rehmanniae Radix, Cinnamomi Cortex, Ephedrae Herba, Cervi Cornu Colla, Sinapis Semen, Zingiberis Rhizoma Preparata, Glycyrrhizae Radix et Rhizoma	Downregulation of IL-6, IL-17A and TNF-α expression.	(75)
	Huatan Quyu Decoction	Bupleuri Radix, Pinelliae Rhizoma, Citri Reticulatae Pericarpium, Aurantii Fructus, Chuanxiong Rhizoma, Atractylodis Macrocephalae Rhizoma, Gleditsiae Spina, Forsythiae Fructus, Scutellariae Radix, Smilacis Glabrae Rhizoma, Salviae Miltiorrhizae Radix et Rhizoma, Paeoniae Radix Rubra, Glycyrrhizae Radix et Rhizoma	Downregulation of NLRP3 inflammasome and IL-1β, IL-18 expression.	(76)
	Yiyi Baijiang Decoction	Paridis Rhizoma, Hedyotidis Herba, Codonopsis Radix, Atractylodis Macrocephalae Rhizoma, Thlaspi arvense Linn, Lonicerae Japonicae Flos, Taxilli Herba, Coicis Semen, Poria, Lobeliae Chinensis Herba, Dipsaci Radix, Moutan Cortex, Violae Herba, Salviae Miltiorrhizae Radix et Rhizoma, Asari Radix et Rhizoma	Downregulation of CRP, IL-4 expression, white blood cell count, and neutrophil levels.	(77)
	Shugan Xiaozhong Powder	Bupleuri Radix, Trichosanthis Fructus, Salviae Miltiorrhizae Radix et Rhizoma, Poria, Curcumae Radix, Bombyx Batryticatus, Hordei Fructus Germinatus, Scutellariae Radix, Cyperi Rhizoma, Myrrha, Gardeniae Fructus, Citri Reticulatae Pericarpium Viride, Citri Reticulatae Pericarpium, Ostreae Concha, Sinapis Semen, Glycyrrhizae Radix et Rhizoma	Upregulation of IgA, IgG, and IgM expression, downregulation of IL-6, CRP, and TNF-α express.	(78)
	Zicao Yanghe Decoction	Arnebiae Radix, Rehmanniae Radix, Cervi Cornu Colla, Zingiberis Rhizoma Preparata, Fritillariae Thunbergii Bulbus, Gleditsiae Spina, Sinapis Semen, Liquidambaris Fructus, Prunellae Spica, Glycyrrhizae Radix et Rhizoma, Ephedrae Herba, Cinnamomi Cortex	Downregulation of GSDMD, caspase-1, NLRP3, IL-18, cleared caspase-1, and cleared IL-1β and GSDMD-N expression.	(79, 80)
	Mahonia bealei	Berberidaceae	Downregulation of IL-1β, IL-6, and CCL5, inhibition of NF-κB and AP-1, phosphorylation of JNK and p38; generation of ROS, NO, iNOS, and COX-2.	(14)
**External treatment methods**	Zicao Yanghe Decoction	ditto	Downregulation of IL-1β, IL-18, caspase-1, GSDMD, and NLRP3 expression.	(10)
	Mahuang Tincture	Strychni Semen, Coptidis Rhizoma, Momordicae Semen, Gardeniae Fructus	Downregulation of CD4^+^expression and CD4^+^/CD8^+^ratio, upregulation of CD8^+^expression.	(81)
	Closed Negative Pressure Drainage Combined with Tori Sputum	Astragali Radix, Angelicae Sinensis Radix, Chuanxiong Rhizoma, Manis, Gleditsiae Spina	Downregulation of IgG4^+^, CD4^+^, CD8^+^, and C1q expression.	(66)
	Shengji Yuhong Ointment	Codonopsis Radix, Chuanxiong Rhizoma, Paeoniae Radix Alba, Astragali Radix, Atractylodis Macrocephalae Rhizoma, Poria, Angelicae Sinensis Radix, Lonicerae Japonicae Flos, Angelicae Dahuricae Radix, Platycodonis Radix, Gleditsiae Spina, Glycyrrhizae Radix et Rhizoma	Downregulation of IL-6, TNF-α expression.	(82)
	Regulating Qi and Ying therapy combined with fire needling cupping	Chuanxiong Rhizoma, Angelicae Sinensis Radix, Rehmanniae Radix, Paeoniae Radix Rubra, Angelicae Dahuricae Radix, Saposhnikoviae Radix, Pinelliae Rhizoma, Fritillariae Thunbergii Bulbus, Salviae Miltiorrhizae Radix et Rhizoma, Lonicerae Japonicae Flos, Taraxaci Herba, Persicae Semen, Carthami Flos, Glycyrrhizae Radix et Rhizoma, Scolopendra	Upregulate the expression of CD3^+^T cells, CD4^+^T cells, and CD8^+^T cells, and downregulate the expression of CD56^+^CD16^+^NK cells, IgM, C3, and C4.	(67)
	Sancai points therapy plus lactiferous ducts perfusion	Qimen, Rugen, Douxue, Xiongxiang, Danzhong, Yingchuang	Downregulation of IL-2, IL-4, and CRP expression.	(83)
**Combined internal and external treatment methods**	Digestive powder combined with modified Yanghe Decoction	modified Yanghe Decoction: Rehmanniae Radix, Cinnamomi Cortex, Cervi Cornu Colla, Sinapis Semen, Zingiberis Rhizoma Preparata, Chuanxiong Rhizoma, Bupleuri Radix, Curcumae Radix; Digestive powder: Zingiberis Rhizoma Recens, Carthami Flos, Cinnamomi Cortex, Sinapis Semen, Ephedrae Herba, Arisaematis Rhizoma, Pinelliae Rhizoma, Aconiti Lateralis Radix Praeparata	Downregulation of CRP, IL-2, IL-4 expression.	(84)
	Xiaoyong Rukang Decoction combined with topical application of Jinhuang ointment	Jinhuang ointment: Trichosanthis Radix, Curcumae Longae Rhizoma, Angelicae Dahuricae Radix, tractylodis Rhizoma, Rhei Radix et Rhizoma, Phellodendri Chinensis Cortex, Plumbum Rubrum; Xiaoyong Rukang Decoction: Bupleuri Radix, Astragali Radix, Salviae Miltiorrhizae Radix et Rhizoma, Forsythiae Fructus, Cyperi Rhizoma, Myrrha, Ranunculus ternatus Thunb, Poria, Scutellariae Radix, Smilacis Glabrae Rhizoma, Gardeniae Fructus, Cremastrae Pseudobulbus Pleiones Pseudobulbus, Taraxaci Herba, Lonicerae Japonicae Flos, Trichosanthis Fructus, Glycyrrhizae Radix et Rhizoma	Upregulation of Treg/Th17 ratio, Treg cells, IL-10, TGF-β1 expression, and downregulation of IgG, IgA levels.	(41)

In TCM, external treatments offer unique advantages and potential applications for managing GLM (**Table 1**). Gao et al. (66) demonstrated that wound irrigation with Tounong Decoction, combined with vacuum sealing drainage, significantly shortened healing times, reduced dressing change frequency, relieved breast abscesses and pain, and promoted granulation tissue growth. Mechanistically, this treatment approach was associated with reductions in serum IgG4^+^, CD4^+^, CD8^+^, and C1q levels. Similarly, Tang et al. (41) reported that Xiaoyong Rukang Decoction, combined with the topical application of Jinhuang ointment, significantly elevated Treg cells, the Treg/Th17 ratio, IL-10, TGF-β1, IgG, and IgA levels. Furthermore, this treatment approach exhibited better safety, a lower recurrence rate, and superior overall efficacy compared to conventional therapies. Acupuncture has also been validated to be an effective immunomodulatory strategy in GLM. Hu et al. (67) found that regulating Qi and Ying, combined with fire needling cupping, enhanced CD3^+^, CD4^+^, and CD8^+^T cells, while decreasing CD56^+^CD16^+^NK cells, IgM, C3, and C4 levels. These changes alleviated mammary tissue degeneration and necrosis, improving both clinical symptoms and immune function in patients with GLM.

**Figure 3:**
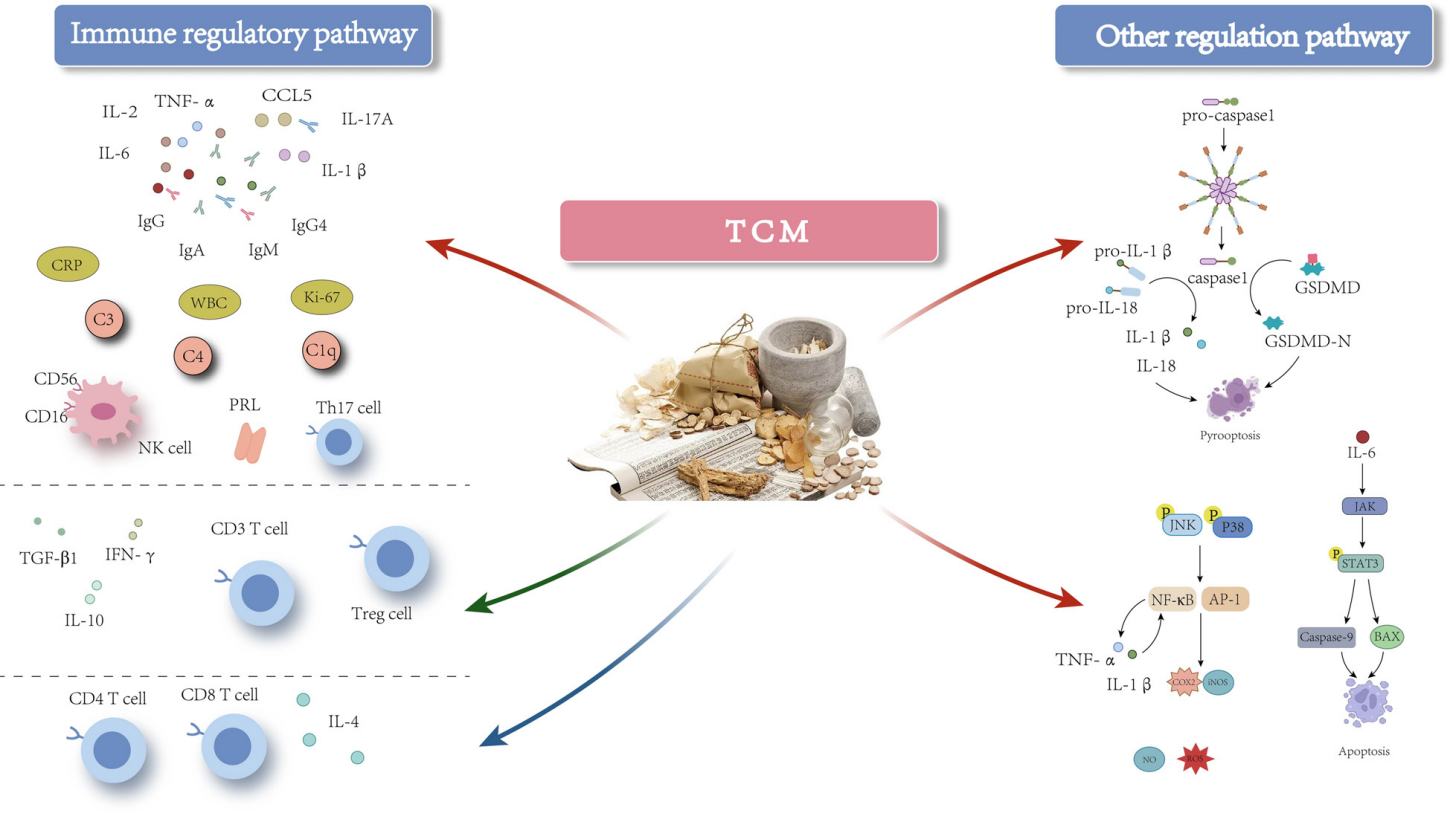
The mechanism of action of TCM-based interventions in treating GLM. Red arrows indicate inhibition, green arrows indicate promotion, and blue arrows indicate either inhibitory or promoting effects at different stages of GLM.

In addition to its immunoregulatory role, TCM can also alleviate inflammatory damage caused by pyroptosis in the treatment of GLM (**Figure 3**). Pyroptosis, a type of inflammatory cell death that occurs when inflammatory stimuli lead to the formation of pores in the cell membrane, primarily mediated by proteins from the Gasdermin (GSDM) family. Intracellular pattern recognition receptors activate the NOD-like receptor protein 3 (NLRP3) inflammasome, which subsequently activates caspase-1. Caspase-1 cleaves GSDMD, leading to pore formation and lysis of the cell membrane, thereby inducing pyroptosis. Furthermore, activated caspase-1 promotes the maturation and release of pro-inflammatory cytokines such as pro-IL-1β and pro-IL-18, exacerbating inflammation (85, 86). Studies have demonstrated that pyroptosis plays a significant role in the pathogenesis of various autoimmune diseases (87–89). Researchers have identified elevated expression levels of pyroptosis-related proteins, including cleaved IL-18, IL-18, cleaved IL-1β, IL-1β, cleaved caspase-1, caspase-1, GSDMD, GSDMD-N, and NLRP3, in GLM tissues. Additionally, transmission electron microscopy has revealed distinct characteristics of pyroptosis at lesion sites, highlighting its involvement in the progression of GLM (10, 79, 90). Oral administration of TCM treatments such as Zicao Yanghe Decoction has been shown to downregulate the expression levels of cleaved IL-18, IL-18, cleaved IL-1β, IL-1β, cleaved caspase-1, caspase-1, GSDMD, GSDMD-N, and NLRP3, thereby inhibiting pyroptosis-mediated inflammation and mitigating GLM-induced inflammatory damage (10, 79). Other formulations, including Tuoli Tounong Powder and Chaihu Qinggan Decoction, have also been found effective in reducing inflammatory damage by targeting the pyroptosis pathway (65, 69). These findings suggest that targeting pyroptosis-mediated inflammation could be a promising therapeutic strategy for managing GLM.

Evidence suggests a close relationship between pyroptosis and immune cells. On one hand, pyroptosis plays a crucial role in modulating the expression of immune cells and shaping the immune microenvironment. For instance, Dong et al. (91), in a study of patients with septic shock, found that pyroptosis leads to the depletion of immature/transitional (IM) B cells, activated memory (AM) B cells, and resting memory (RM) B cells in peripheral blood. The administration of the caspase-1 inhibitor VX-765 effectively mitigated the depletion of these B cell subsets. Similarly, Zhang et al. (92) demonstrated that GSDME expression enhances the phagocytic capacity of tumor-associated macrophages (TAMs) and promotes the infiltration of NK and CD8^+^T cells into tumors, thereby strengthening adaptive anti-tumor immunity. Furthermore, granzyme (Gzm) B from cytotoxic cells can directly cleave GSDME to induce caspase-independent pyroptosis in target cells, thereby modulating the activation of immune cell populations. On the other hand, cytokines released during pyroptosis, such as IL-18, significantly enhance the cytotoxic functions of T cells and NK cells, while also stimulating IFN-γ secretion from T cells, which encourages M1 polarization of TAMs (93). Additionally, IL-18 can promote the secretion of GM-CSF and other cytokines, thereby facilitating the differentiation of monocytes into M1 macrophages. Additionally, immune cell functionality is closely linked to pyroptosis. Research shows that GzmA, stored in the lytic granules of CTLs and NK cells, regulates pyroptosis in GSDMB-positive cells. Specifically, GzmA cleaves GSDMB at Lys^244^, leading to the release of its pore-forming protein perforin, which induces pyroptotic cell death in targeted cells. Activated CTLs can further amplify GzmA-mediated pyroptotic cell death by enhancing GSDMB expression through the release of IFN-γ (94). Further investigations revealed that cells expressing GSDMB3 undergo pyroptosis via membrane pore formation following NK cell attack, whereas cells expressing GSDMB4 partially resist NK cell-induced lysis, exhibiting a mixed phenotype characterized by both pyroptosis and apoptosis (95).

Based on the aforementioned findings, we hypothesize that pyroptosis may play a critical role in the pathogenesis of GLM by modulating the immune system, thereby influencing its progression and prognosis. Studies have demonstrated a significant upregulation in the expression of apoptosis-related proteins, such as Caspase-9 and Bcl-2-associated X protein (BAX), in GLM, potentially exacerbating the inflammatory response. TCM, specifically the Xihuang capsule, has shown efficacy in inhibiting excessive apoptosis in breast tissue, thereby alleviating inflammation and reducing tissue damage (71). Additionally, pyroptosis is a form of regulated cell death (RCD) that does not act independently but interacts with other RCD processes, including ferroptosis, autophagy, and apoptosis, to varying degrees (96). Consequently, it is essential to investigate whether these alternative forms of RCD contribute to the progression of GLM or mediate inflammation and immune dysregulation through immune pathways. Meanwhile, TCM, characterized by its multi-component, multi-pathway, and multi-target action, plays a role in immunomodulation and the regulation of pyroptosis in treating GLM. Various Chinese medicine compounds, such as Shengji Yuhong Ointment and Tounong Powder, have demonstrated precise therapeutic effects in modulating immune cell responses and cytokine-mediated injuries associated with GLM. However, it remains to be elucidated whether these compounds exert their therapeutic effects on GLM through the pyroptosis pathway and whether the herbal ingredients involved in regulating pyroptosis also possess immunomodulatory effects. Further research is necessary to clarify these mechanisms and establish more effective treatment strategies.

## 4 Conclusion

In summary, GLM is classified as an autoimmune disease involving a variety of immune cells and cytokines. Current research indicates that TCM formulations, such as Chaihu Qinggan Decoction and Yiqi Heying Decoction, along with therapies like fire needle and cupping, can effectively suppress immune-mediated inflammatory damage through immunomodulatory mechanisms, leading to therapeutic benefits. Furthermore, we found that TCM treatment for GLM not only regulates the immune system but also modulates pyroptosis to mitigate inflammatory damage. Herbal constituents and formulations, including Mahonia bealei and Chaihu Qinggan Decoction, facilitate pyroptosis while exerting immunomodulatory effects to counteract inflammation during GLM progression. Further studies have revealed that pyroptosis not only participates in the pathological damage associated with GLM but also interacts with various immune cells and inflammatory factors, including T cells, B cells, and IL-18. The interplay among these components influences both tissue injury and prognosis in GLM. The complex regulatory network of RCD provides a new avenue for understanding the pathogenesis of GLM: investigating the crosstalk between pyroptosis and other forms of RCD could reveal additional insights into their roles in the disease process; at the same time, in-depth exploration of the interaction mechanism between the regulatory network of RCD and that of the immune system, is expected to provide more precise therapeutic approaches for the treatment of GLM. Finally, further exploration of the therapeutic role of TCM in GLM in order to identify its multi-component, multi-pathway, and multi-target action characteristics, and to provide guidance for the compounding of TCM-based formulations and the selection of meridians and acupoints for acupuncture will be conducive to a more comprehensive investigation of the pathogenesis of GLM and to the development of more effective therapeutic approaches.

We also recognize that, in the face of the complex pathogenic mechanisms of GLM, there are differences in cytokine- and immune cell-mediated pathogenic mechanisms in different pathological stages, different medical histories, and different detection sites (peripheral blood and focal tissues). Studies based on the complement system and autoantibodies in the pathogenic mechanisms of GLM are slightly insufficient and controversial. Meanwhile, compared with the complex pathological injury mechanism of GLM, the number of mechanistic studies on the use of TCM-based interventions for managing GLM is relatively small, and most of the studies are based on clinical experiments, and there is a relative lack of comprehensive animal and cellular experiments. With the deepening of the research, more RCD- and immune system-related targets and pathways have been identified, although only a small portion has been validated in GLM, and even fewer studies have involved TCM-based treatment strategies. Therefore, in order to better understand the exact mechanism of action of TCM-based interventions, it is necessary for researchers to employ modern biotechnological tools to more comprehensively analyze the pathogenic mechanism of GLM and provide a more precise basis for individualized treatment, which is also the future direction of development to promote the modernization of TCM-based interventions and give full play to its own advantages.

## References

[1] Musleh A Shrateh ON Ishtaya N Abbadi K Asbah M Ayyad S. A single center experience with a rare clinical entity of idiopathic granulomatous mastitis: Case series and review of the literature. Int J Surg Case Rep. (2024) 115:109232. doi: 10.1016/j.ijscr.2024.109232 38217923 PMC10826802

[2] Yuan QQ Xiao SY Farouk O Du YT Sheybani F Tan QT. Management of granulomatous lobular mastitis: an international multidisciplinary consensus (2021 edition). Mil Med Res. (2022) 9:20. doi: 10.1186/s40779-022-00380-5 35473758 PMC9040252

[3] Fattahi AS Amini G Sajedi F Mehrad-Majd H. Factors affecting recurrence of idiopathic granulomatous mastitis: A systematic review. Breast J. (2023) 2023:9947797. doi: 10.1155/2023/9947797 37794976 PMC10547579

[4] Papila Kundaktepe B Velidedeoglu M Mete B. The effect of methotrexate monotherapy on treatment-resistant idiopathic granulomatous mastitis patients. Surgeon. (2022) 20:e13–9. doi: 10.1016/j.surge.2021.03.001 33836950

[5] Wang X He X Liu J Zhang H Wan H Luo J. Immune pathogenesis of idiopathic granulomatous mastitis: from etiology toward therapeutic approaches. Front Immunol. (2024) 15:1295759. doi: 10.3389/fimmu.2024.1295759 38529282 PMC10961981

[6] Zuo X Shi X Gao X Lai R Liu P Zhao Z. A retrospective study on 221 patients with granulomatous lobular mastitis treated by a combination of traditional Chinese medicine and western medicine. Ann Ital Chir. (2021) 92:135–41. 34031280

[7] Zhu Q Liu XF Wang N Wang N Zhang Y Song AL. Clinical experience of professor song aili in the treatment of granulomatous mastitis. Asia-Pacific Traditional Med. (2019) 15:87–9. doi: 10.11954/ytctyy.201903028

[8] Guo JR Sun CP Liu S. Research progress of the TCM treatment for granulomatous lobular mastitis. Chin J Library Inf Sci Traditional Chin Med. (2024) 48:248–51. doi: 10.3969/j.issn.2095-5707.202211070

[9] Wang Y Fan HQ Ge AQ Ge AQ Zhou L. Clinical observation on Chaihu Qinggan Decoction combined with prednisone on granulomatous mastitis and its impact on immune cell function. J Hubei Univ Chin Med. (2023) 25:57–9. doi: 10.3969/j.issn.1008-987x.2023.04.15

[10] Mu YJ Wang TS Feng X Sun P Gao S Wang YK. Clinical eficacy of external treatment with Zi Cao Yang He Tang combined with hormone therapy for granulomatous mastitis and its effect on celular pyroptosis proteins. J Hainan Med Univ. (2023) 29:1703–1709+1717. doi: 10.13210/j.cnki.jhmu.20230928.003

[11] Liu XF Wang YY Su QQ Li JW Chen HH Zhang LM. Regulating the lmmune lmbalance lechanism of IL-6 and IL-1a、IFN-a in Granulomatous Mastitis by Yigi Heying Method. Modernization Traditional Chin Med Materia Medica-World Sci Technol. (2022) 24:3442–8. doi: 10.11842/wst.20210914001

[12] Wilson JL Mayr HK Weichhart T. Metabolic programming of macrophages: implications in the pathogenesis of granulomatous disease. Front Immunol. (2019) 10:2265. doi: 10.3389/fimmu.2019.02265 31681260 PMC6797840

[13] Xing Z Afkhami S Bavananthasivam J Fritz DK D'Agostino MR Vaseghi-Shanjani M. Innate immune memory of tissue-resident macrophages and trained innate immunity: Re-vamping vaccine concept and strategies. J Leukoc Biol. (2020) 108:825–34. doi: 10.1002/JLB.4MR0220-446R 32125045

[14] Wang Z Wang N Liu X Wang Q Xu B Liu P. Broadleaf Mahonia attenuates granulomatous lobular mastitis−associated inflammation by inhibiting CCL−5 expression in macrophages. Int J Mol Med. (2018) 41:340–52. doi: 10.3892/ijmm.2017.3246 PMC5746325 29138800

[15] Huang YM Lo C Cheng CF Lu CH Hsieh SC Li KJ. Serum C-reactive protein and interleukin-6 levels as biomarkers for disease severity and clinical outcomes in patients with idiopathic granulomatous mastitis. J Clin Med. (2021) 10(10):2077. doi: 10.3390/jcm10102077 PMC8150275 34066203

[16] de la Calle-Fabregat C Calafell-Segura J Gardet M Dunsmore G Mulder K Ciudad L. NF-kappaB and TET2 promote macrophage reprogramming in hypoxia that overrides the immunosuppressive effects of the tumor microenvironment. Sci Adv. (2024) 10:eadq5226. doi: 10.1126/sciadv.adq5226 39292770 PMC11409945

[17] Chen DP Wang JC Liu ZY Li PL Chan KW Wu XN. miRNome targeting NF-kappaB signaling orchestrates macrophage-triggered cancer metastasis and recurrence. Mol Ther. (2024) 32:1110–24. doi: 10.1016/j.ymthe.2024.02.009 PMC11163221 38341612

[18] Mosser DM Hamidzadeh K Goncalves R. Macrophages and the maintenance of homeostasis. Cell Mol Immunol. (2021) 18:579–87. doi: 10.1038/s41423-020-00541-3 PMC7491045 32934339

[19] Hu Q Lyon CJ Fletcher JK Tang W Wan M Hu TY. Extracellular vesicle activities regulating macrophage- and tissue-mediated injury and repair responses. Acta Pharm Sin B. (2021) 11:1493–512. doi: 10.1016/j.apsb.2020.12.014 PMC8245807 34221864

[20] Kong C Zhang C Wu Y Zeng Z Yu H Zeng J. The expression and meaning of CD68, CD163, CD57, and IgG4 in granulomatous lobular mastitis. Gland Surg. (2020) 9:936–49. doi: 10.21037/gs-20-419 PMC7475371 32953603

[21] Zheng B Song J Lu M Chen C Sun S. Current research describing the role of CD4(+) T lymphocyte subsets in the pathogenesis of granulomatous lobular mastitis. J Invest Surg. (2022) 35:1790–5. doi: 10.1080/08941939.2022.2090035 36075587

[22] Li Y Chen L Zhang C Wang Y Hu J Zhou M. Clinicopathologic features and pathogens of granulomatous lobular mastitis. Breast Care (Basel). (2023) 18:130–40. doi: 10.1159/000529391 PMC10228255 37261131

[23] Koksal H Vatansev H Artac H Kadoglou N. The clinical value of interleukins-8, -10, and -17 in idiopathic granulomatous mastitis. Clin Rheumatol. (2020) 39:1671–7. doi: 10.1007/s10067-020-04925-8 31916110

[24] Saydam M Yilmaz KB Sahin M Yanik H Akinci M Yilmaz I. New findings on autoimmune etiology of idiopathic granulomatous mastitis: serum IL-17, IL-22 and IL-23 levels of patients. J Invest Surg. (2021) 34:993–7. doi: 10.1080/08941939.2020.1725190 32046543

[25] Yang XH. The relative study of expression ofIL-2, 1L-4 in the tissue of GM. Jinan City, Shandong Province, China: Shandong University of Traditional Chinese Medicine (2012).

[26] Kokubo K Onodera A Kiuchi M Tsuji K Hirahara K Nakayama T. Conventional and pathogenic Th2 cells in inflammation, tissue repair, and fibrosis. Front Immunol. (2022) 13:945063. doi: 10.3389/fimmu.2022.945063 36016937 PMC9395650

[27] Hirahara K Shinoda K Morimoto Y Kiuchi M Aoki A Kumagai J. Immune cell-epithelial/mesenchymal interaction contributing to allergic airway inflammation associated pathology. Front Immunol. (2019) 10:570. doi: 10.3389/fimmu.2019.00570 30972065 PMC6443630

[28] Mitson-Salazar A Yin Y Wansley DL Young M Bolan H Arceo S. Hematopoietic prostaglandin D synthase defines a proeosinophilic pathogenic effector human T(H)2 cell subpopulation with enhanced function. J Allergy Clin Immunol. (2016) 137:907–918.e909. doi: 10.1016/j.jaci.2015.08.007 26431580

[29] Wang MX Wang AD Yang WX Liu BS. Study on the drug serum of Xilao Dihuang Combined Prescription regulating Teff/Treg differentiation and function in ITP patients. Tianjin Med J. (2022) 50:487–92. doi: 10.11958/20212724

[30] Legorreta-Haquet MV Chavez-Rueda K Chavez-Sanchez L Cervera-Castillo H Zenteno-Galindo E Barile-Fabris L. Function of treg cells decreased in patients with systemic lupus erythematosus due to the effect of prolactin. Med (Baltimore). (2016) 95:e2384. doi: 10.1097/MD.0000000000002384 PMC4748869 26844452

[31] Ucaryilmaz H Koksal H Emsen A Kadoglou N Dixon JM Artac H. The role of regulatory T and B cells in the etiopathogenesis of idiopathic granulomatous mastitis. Immunol Invest. (2022) 51:357–67. doi: 10.1080/08820139.2020.1832114 33034215

[32] Jia Q Hu J Wang X Deng Y Zhang J Li H. Malassezia globosa induces differentiation of pathogenic th17 cells by inducing IL-23 secretion by keratinocytes. Mycopathologia. (2024) 189:85. doi: 10.1007/s11046-024-00890-x 39283337

[33] Legorreta-Haquet MV Santana-Sanchez P Chavez-Sanchez L Chavez-Rueda AK. The effect of prolactin on immune cell subsets involved in SLE pathogenesis. Front Immunol. (2022) 13:1016427. doi: 10.3389/fimmu.2022.1016427 36389803 PMC9650038

[34] Ewerman L Landberg E Hellberg S Hovland M Sundin A Jenmalm MC. Immunomodulating effects depend on prolactin levels in patients with hyperprolactinemia. Horm Metab Res. (2020) 52:228–35. 10.1055/a-1126-4272 32268424

[35] Kobayashi T Kawamura H Kanda Y Matsumoto H Saito S Takeda K. Natural killer T cells suppress zymosan A-mediated granuloma formation in the liver by modulating interferon-gamma and interleukin-10. Immunology. (2012) 136:86–95. doi: 10.1111/j.1365-2567.2012.03562.x 22268994 PMC3372760

[36] Emsen A Koksal H Ucaryilmaz H Kadoglou N Artac H. The alteration of lymphocyte subsets in idiopathic granulomatous mastitis. Turk J Med Sci. (2021) 51:1905–11. doi: 10.3906/sag-2012-192 PMC8569769 33862673

[37] Lee DSW Rojas OL Gommerman JL. B cell depletion therapies in autoimmune disease: advances and mechanistic insights. Nat Rev Drug Discovery. (2021) 20:179–99. doi: 10.1038/s41573-020-00092-2 PMC7737718 33324003

[38] Rawlings DJ Metzler G Wray-Dutra M Jackson SW. Altered B cell signalling in autoimmunity. Nat Rev Immunol. (2017) 17:421–36. doi: 10.1038/nri.2017.24 PMC5523822 28393923

[39] Jansen K Cevhertas L Ma S Satitsuksanoa P Akdis M van de Veen W. Regulatory B cells, A to Z. Allergy. (2021) 76:2699–715. doi: 10.1111/all.14763 33544905

[40] Catalan D Mansilla MA Ferrier A Soto L Oleinika K Aguillon JC. Immunosuppressive mechanisms of regulatory B cells. Front Immunol. (2021) 12:611795. doi: 10.3389/fimmu.2021.611795 33995344 PMC8118522

[41] Tang Z Ling J Huang WF. Therapeutic Efects of Xiaoyong Rukang Decoction Combined with Herbal Dressing Change on Granulomatous Mastitis and its influence on Treg/Th17 lmmune Balance Mechanism. Western J Traditional Chin Med. (2023) 36:145–9. doi: 10.12174/j.issn.2096-9600.2023.10.31

[42] Fang XF Liu XF Song AL Li SY. Clinical Optimization scheme and lmmune lntervention Mechanism of Minimally invasive Debridement Combined with Yigi Heying Chinese Medicinal in Treatment of GM. Inf Traditional Chin Med. (2023) 40:58–63+68. doi: 10.19656/j.cnki.1002-2406.20230509

[43] Ogura K Matsumoto T Aoki Y Kitabatake T Fujisawa M Kojima. IgG4-related tumour-forming mastitis with histological appearances of granulomatous lobular mastitis: comparison with other types of tumour-forming mastitis. Histopathology. (2010) 57:39–45. doi: 10.1111/j.1365-2559.2010.03581.x 20653779

[44] Lopez-Vergès S Milush JM Pandey S York VA Arakawa-Hoyt J Pircher H. CD57 defines a functionally distinct population of mature NK cells in the human CD56dimCD16+ NK-cell subset. Blood. (2010) 116:3865–74. doi: 10.1182/blood-2010-04-282301 PMC2981540 20733159

[45] Deng Y Kerdiles Y Chu J Yuan S Wang Y Chen X. Transcription factor Foxo1 is a negative regulator of natural killer cell maturation and function. Immunity. (2015) 42:457–70. doi: 10.1016/j.immuni.2015.02.006 PMC4400836 25769609

[46] Tufa DM Shank T Yingst AM Trahan GD Shim S Lake J. Prolactin acts on myeloid progenitors to modulate SMAD7 expression and enhance hematopoietic stem cell differentiation into the NK cell lineage. Sci Rep. (2020) 10:6335. doi: 10.1038/s41598-020-63346-4 32286456 PMC7156717

[47] Godoy-Pacheco A Garcia-Chagollan M Ramirez-De-Arellano A Hernandez-Silva CD Villegas-Pineda JC Ramirez-Lopez IG. Differential modulation of natural killer cell cytotoxicity by 17beta-estradiol and prolactin through the NKG2D/NKG2DL axis in cervical cancer cells. Oncol Lett. (2022) 24:288. doi: 288. doi 35814823 10.3892/ol.2022.13408 PMC9260731

[48] Su QQ Liu XF Li FF Li JW Chen HH Wang FY. Clinical efect of yigi heving formula combined with hormone therapy in treating IGM and mechanism of C3 and C4 lmmune disorde. Acta Chin Med Pharmacol. (2022) 50:48–53. doi: 10.19664/j.cnki.1002-2392.220181

[49] Li XQ Sun HG Wang XH Zhang HJ Zhang XS Yu Y. Activation of C3 and C5 may be involved in the inflammatory progression of PCM and GM. Inflammation. (2022) 45:739–52. doi: 10.1007/s10753-021-01580-2 34997873

[50] Bouwman HB Guchelaar HJ. The efficacy and safety of eculizumab in patients and the role of C5 polymorphisms. Drug Discovery Today. (2024) 29:104134. doi: 10.1016/j.drudis.2024.104134 39111540

[51] Atanes P Ruz-Maldonado I Pingitore A Hawkes R Liu B Zhao M. C3aR and C5aR1 act as key regulators of human and mouse beta-cell function. Cell Mol Life Sci. (2018) 75:715–26. doi: 10.1007/s00018-017-2655-1 PMC5769825 28921001

[52] Ozel L Unal A Unal E Kara M Erdogdu E Krand O. Granulomatous mastitis: is it an autoimmune disease? Diagnostic and therapeutic dilemmas. Surg Today. (2012) 42:729–33. doi: 10.1007/s00595-011-0046-z 22068681

[53] Shojaeian F Haghighat S Abbasvandi F Houshdar Tehrani A Najar Najafi N Zandi A. Refractory and recurrent idiopathic granulomatous mastitis treatment: adaptive, randomized clinical trial. J Am Coll Surg. (2024) 238(6):1153–65. doi: 10.1097/XCS.0000000000001046 38372343

[54] Koksal H. The clinical utility of autoantibodies in patients with idiopathic granulomatous mastitis. J Invest Surg. (2022) 35:325–9. doi: 10.1080/08941939.2020.1861666 33327830

[55] Ma F Cao Y Du H Braikia FZ Zong L Ollikainen N. Three-dimensional chromatin reorganization regulates B cell development during ageing. Nat Cell Biol. (2024) 26:991–1002. doi: 10.1038/s41556-024-01424-9 38866970 PMC11178499

[56] Mattos MS Vandendriessche S Waisman A Marques PE. The immunology of B-1 cells: from development to aging. Immun Ageing. (2024) 21:54. doi: 10.1186/s12979-024-00455-y 39095816 PMC11295433

[57] de Mol J Kuiper J Tsiantoulas D Foks AC. The dynamics of B cell aging in health and disease. Front Immunol. (2021) 12:733566. doi: 10.3389/fimmu.2021.733566 34675924 PMC8524000

[58] Silva D Silva MVD Barros CCO Alexandre PBD Timoteo RP Catarino JS. TNF-alpha blockade impairs in *vitro* tuberculous granuloma formation and down modulate Th1, Th17 and Treg cytokines. PloS One. (2018) 13:e0194430. doi: 10.1371/journal.pone.0194430 29543912 PMC5854376

[59] Liu XF Wang N Li FF Song AL Zhang LM. Expressions and Clinical significance of lmmune-Related factors in Diferent TCM Syndrome Patterns of Granulomatous Mastitis. Acta Chin Med Pharmacol. (2020) 48:23–8. doi: 10.19664/j.cnki.1002-2392.200063

[60] Diao Y Shan CY Zhao Y Jin YF Zhang SQ Guan HT. Role of lL-6/STAT3 signaling pathway in the granulomatous mastitis. Prog Modern Biomedicine. (2018) 18:4486–4488+4438. doi: 10.13241/j.cnki.pmb.2018.23.020

[61] Boutet P Sulon J Closset R Detilleux J Beckers JF Bureau F. Prolactin-induced activation of nuclear factor kappaB in bovine mammary epithelial cells: role in chronic mastitis. J Dairy Sci. (2007) 90:155–64. doi: 10.3168/jds.S0022-0302(07)72617-6 17183084

[62] Lian WJ Sun YH Zhao PL Sun ZY. Prescription regularity and mechanisms of TCM medicine in treatment of granulomatous lobular mastitis: a study based on data mining and network pharmacology. Clin J Chin Med. (2024) 16:1–8+18. doi: 10.3969/j.issn.1674-7860.2024.01.001

[63] Xie L Wan H Wu XQ Feng JM Gao QQ Qu WC. A study on the mechanism of Chaihu Qinggan Decoction in treating granulomatous mastitis based on network pharmacology and molecular docking. China Med Pharm. (2023) 13:30–4. doi: 10.20116/j.issn2095-0616.2023.23.07

[64] Wu SS Liu CQ Xu Y Bao LJ Gu RH. Clinical efficacy observation of Shugan Xiaoyong decoction and antibiotics on granulomatous mastitis in the period of lump. Shanxi J Traditional Chin Med. (2022) 38:30–2. doi: 10.20002/j.issn.1000-7156.2022.01.010

[65] Zhao Z Zuo XM Wang TS Liu JL Yang ZR Gao S. Effect of Tuoli Tounong Decoction on caspase-1/GSDMD signaling pathway in granulomatous lobular mastitis. Global Traditional Chin Med. (2022) 15:1537–42. doi: 10.3969/j.issn.1674-1749.2022.09.003

[66] Gao X Shi XG Zhou KX Xin M Gao S Zuo XM. Cinical study of closed negative pressure drainage combined with tori soutum external treatment for granulomatous lobular mastitis in abscess stage. Chin J Surg Integrated Traditional Western Med. (2020) 26:476–80. doi: 10.3969/j.issn.1007-6948.2020.03.014

[67] Hu YC Lei QM Ouyang QW. Observation on the effect of regulating qi and ying therapy combined with fire needling cupping in the treatment of granulomatous mastitis. Med Innovation China. (2023) 20:115–8. doi: 10.3969/j.issn.1674-4985.2023.35.026

[68] Wang Y Zhou L Sun T Liu LF. Observation on the curative effect of method for soothing the liver and regulating Qi, removing blood stasis and dredging collaterals on granulomatous mastitis at mass stage. Modern J Integrated Traditional Chin Western Med. (2021) 30:2550–4. doi: 10.3969/j.issn.1008-8849.2021.23.009

[69] Zhou Y Liu LF Liu JL Gong J Liu SL Zhao D. Mechanism of chaihu qinggantang in intervening NLRP3/lL-1β Pathway to treat granulomatous lobular mastitis in rat model. Chin J Exp Traditional Med Formulae. (2022) 28:1–7. doi: 10.13422/j.cnki.syfjx.20221005

[70] Ma LN. Construction of a rat model for Granulomatous Mastitis and the Intervention Mechanism of Chaihu Qinggan Decoction. Jinan City, Shandong Province, China: Shanghai University of Traditional Chinese Medicine (2021).

[71] Wang Y Liu LF Zhou L Hu JH. Mechanism of action for Xihuang Capsule in promoting wound healing in non-lactational mastitis rat model by regulating lL-6/JAK2/STAT3 signaling pathway. J Hunan Univ Chin Med. (2023) 43:1173–9. doi: 10.3969/j.issn.1674-070X.2023.07.004

[72] Dai X Xie MY. Exploration of the mechanism of Xihuang Capsules in the treatment of granulomatous mastitis based on network pharmacology and experimental verification. Chin J Hosp Pharm. (2022) 42:889–95. doi: 10.13286/j.1001-5213.2022.09.03

[73] Xie MY dai X. Observation of the clinical effect of Tounong Powder in the treatment of granulomatous mastitis. Shenzhen J Integrated Traditional Chin Western Med. (2021) 31:58–60. doi: 10.16458/j.cnki.1007-0893.2021.11.025

[74] Liu CY Liu XF Sun Y Chen HH Zhang LM. IL-6 centered immune-inflammatory regulation mechanism of san in intervening GLM based on network pharmacology and molecularTounong docking. Inf Traditional Chin Med. (2023) 40:43–51. doi: 10.19656/j.cnki.1002-2406.20230107

[75] Quan YJ. Clinical observation of Yanghe Decoction in the treatment of helcosis of granulomatous mastitis and the effect on immune inflammatory response. Jinan City, Shandong Province, China: Shandong University of Traditional Chinese Medicine (2023).

[76] Xiong WH. Intervention effect and curative effect of removing phlegm and removing blood stasis method on serum NLRP3 inflammasome, IL-1β and IL-18 in patients with granulomatous mastitis. Hefei City, Anhui Province, China: Anhui University of Traditional Chinese Medicine (2023).

[77] Wang MX Yuan HY. Effects of yiyi baijiang decoction on leukocyte count, neutrophil and CRP in patients with early stage granuloma mastitis. Jilin J Chin Med. (2019) 39:354–6. doi: 10.13463/j.cnki.jlzyy.2019.03.022

[78] Qiu GC Leng J Wan X Xu KQ. Shugan Xiaozhong Powder for patients with granulomatous mastitis of liver depression and phlegm coagulation:curative effect observation and mechanism. Hebei J Traditional Chin Med. (2021) 43:1123–1126+1131. doi: 10.3969/j.issn.1002-2619.2021.07.016

[79] Liu JL Shi XG Wang TS Zuo XM Feng X Wang YK. Effect of zicao yanghe decoction on caspase-1/GSDMD/lL-1β and estrogen and progesterone receptors in granulomatous lobular mastitis. World J Integrated Traditional Western Med. (2022) 17:1551–7. doi: 10.13935/j.cnki.sjzx.220811

[80] Mu YJ Wang TS Feng X Sun P Gao S Wang YK. Clinical efficacy of external treatment with Zi Cao Yang He Tang combined with hormone therapy for granulomatous mastitis and its effect on cellular pyroptosis proteins. J Hainan Med Univ. (2023) 29:1703–1709+1717. doi: 10.13210/j.cnki.jhmu.20230928.003

[81] Wang T. To Explore the Therapeutic Mechanism of Mahuang Tincturein the Treatment of Granulomatous Mammary Glands with Hot,Poisonous and Blazing Syndrome Based on T Lymphocyte Expression. Jinan City, Shandong Province, China: Shandong University of Traditional Chinese Medicine (2022).

[82] Chen TC. Clinical Observation of Shengji Yuhong Ointment for ExternalUse in Treating Granulomatous Mastitis at the Late Stage of Collapse. Jinan City, Shandong Province, China: Shandong University of Traditional Chinese Medicine (2023).

[83] Wen GQ Wu SY Li GH. Application of Sancai points therapy plus lactiferous ducts perfusion on granulomatous mastitis. Clin J Chin Med. (2021) 13:51–4. doi: 10.3969/j.issn.1674-7860.2021.16.015

[84] Liu LH Hou XQ Li QH Ma YH. Clinical observation of internal and external administration of Chinese medicines in the treatment of granulomatous mastitis at mass stage. Pract Pharm Clin Remedies. (2021) 24:342–4. doi: 10.14053/j.cnki.ppcr.202104012

[85] Yu P Zhang X Liu N Tang L Peng C Chen X. Pyroptosis: mechanisms and diseases. Signal Transduct Target Ther. (2021) 6:128. doi: 10.1038/s41392-021-00507-5 33776057 PMC8005494

[86] Kovacs SB Miao EA. Gasdermins: effectors of pyroptosis. Trends Cell Biol. (2017) 27:673–84. doi: 10.1016/j.tcb.2017.05.005 PMC5565696 28619472

[87] You R He X Zeng Z Zhan Y Xiao Y Xiao R. Pyroptosis and its role in autoimmune disease: A potential therapeutic target. Front Immunol. (2022) 13:841732. doi: 10.3389/fimmu.2022.841732 35693810 PMC9174462

[88] Wu XY Li KT Yang HX Yang B Lu X Zhao LD. Complement C1q synergizes with PTX3 in promoting NLRP3 inflammasome over-activation and pyroptosis in rheumatoid arthritis. J Autoimmun. (2020) 106:102336. doi: 10.1016/j.jaut.2019.102336 31601476

[89] Chen X Liu G Yuan Y Wu G Wang S Yuan L. NEK7 interacts with NLRP3 to modulate the pyroptosis in inflammatory bowel disease via NF-kappaB signaling. Cell Death Dis. (2019) 10:906. doi: 10.1038/s41419-019-2157-1 31787755 PMC6885517

[90] Zuo XM Wang TS Shi XG Gao X Gao S Sun P. Pyroptosis: the pathological process that dominates granulomatous lobular mastitis. J Physiol Pharmacol. (2021) 72(23):10. doi: 10.26402/jpp.2021.3.15 34873070

[91] Dong X Tu H Bai X Qin S Li Z. Intrinsic/extrinsic apoptosis and pyroptosis contribute to the selective depletion of B cell subsets in septic shock patients. Shock. (2023) 60:345–53. doi: 10.1097/SHK.0000000000002174 PMC10510799 37477437

[92] Zhang Z Zhang Y Xia S Kong Q Li S Liu X. Gasdermin E suppresses tumour growth by activating anti-tumour immunity. Nature. (2020) 579:415–20. doi: 10.1038/s41586-020-2071-9 PMC7123794 32188940

[93] Tang K Huang N Tan QY. Research progress in pyroptosis, drug therapy and lmmunotherapy in glioblastoma. Chin J Modern Appl Pharm. (2024) 41:287–94. doi: 10.13748/j.cnki.issn1007-7693.20232189

[94] Zhou Z He H Wang K Shi X Wang Y Su Y. Granzyme A from cytotoxic lymphocytes cleaves GSDMB to trigger pyroptosis in target cells. Science. (2020) 368(6494):eaaz7548. doi: 10.1126/science.aaz7548 32299851

[95] Kong Q Xia S Pan X Ye K Li Z Li H. Alternative splicing of GSDMB modulates killer lymphocyte-triggered pyroptosis. Sci Immunol. (2023) 8:eadg3196. doi: 10.1126/sciimmunol.adg3196 37115914 PMC10338320

[96] Lou Y Ma M Jiang Y Xu H Gao Z Gao L. Ferroptosis: A new strategy for traditional Chinese medicine treatment of stroke. BioMed Pharmacother. (2022) 156:113806. doi: 10.1016/j.biopha.2022.113806 36228377

